# MutSα expression predicts a lower disease-free survival in malignant salivary gland tumors: an immunohistochemical study

**DOI:** 10.4317/medoral.25138

**Published:** 2022-02-20

**Authors:** Gleyson Kleber do Amaral-Silva, Laryssa Moura Dias, Bruno Augusto Linhares Almeida Mariz, Felipe Paiva Fonseca, Ana Lúcia Carrinho Ayroza Rangel, Virgílio Gonzales Zanella, Rogerio Moraes Castilho, Manoela Domingues Martins, Pablo Agustin Vargas, Vivian Petersen Wagner

**Affiliations:** 1 Department of Oral Diagnosis, Piracicaba Dental School, University of Campinas, Piracicaba, SP, Brazil; 2 Department of Oral Surgery and Pathology, School of Dentistry, Universidade Federal de Minas Gerais, Belo Horizonte, MG, Brazil; 3 Department of Pathology, State University of Western Paraná, Cascavel, PR, Brazil; 4 Head and Neck Surgery Department, Santa Rita Hospital, Santa Casa de Misericórdia de Porto Alegre, Porto Alegre, RS, Brazil; 5 Laboratory of Epithelial Biology, Department of Periodontics and Oral Medicine, University of Michigan School of Dentistry, Ann Arbor, MI, USA; 6 Department of Pathology, School of Dentistry, Federal University of Rio Grande do Sul, Porto Alegre, RS, Brazil; 7 Department of Oral Pathology and Oral Biology, School of Dentistry, University of Pretoria, Pretoria, South Africa; 8 Academic Unit of Oral and Maxillofacial Medicine and Pathology, Department of Clinical Dentistry, University of Sheffield, Sheffield, UK

## Abstract

**Background:**

Appropriate DNA replication is vital to maintain cell integrity at the genomic level. Malfunction on DNA repair mechanisms can have implications related to tumor behavior. Our aim was to evaluate the expression of key complexes of the DNA mismatch-repair system MutSα (hMSH2-hMSH6) and MutSβ (hMSH2-hMSH3) in a panel comprising the most common benign and malignant salivary gland tumors (SGT), and to determine their association with disease-free survival.

**Material and Methods:**

Ten cases of normal salivary gland (NSG) and 92 of SGT (54 benign and 38 malignant) were retrieved. Immunohistochemistry was performed for hMSH2, hMSH3, hMSH6. Scanned slides were digitally analyzed based on the percentage of positive cells with nuclear staining. Cases were further classified in MutSαhigh and MutSβhigh based on hMSH2-hMSH6 and hMSH3-hMSH6 expression, respectively.

**Results:**

hMSH3 expression was lower in malignant SGT compared to NSG and benign cases. Adenoid cystic carcinoma (ACC) cases with perineural invasion presented a lower percentage of hMSH3 positive cells. hMSH6 was downregulated in both benign and malignant SGT compared to NSG. Malignant SGT cases with MutSαhigh expression had lower disease-free survival compared to MutSαlow cases. A 10.26-fold increased risk of presenting local recurrence was observed.

**Conclusions:**

Our findings suggest that a lack of hMSH3 protein function is associated with a more aggressive phenotype (malignancy and perineural invasion) and that MutSα overexpression predicts a poor clinical outcome in malignant SGT.

** Key words:**Salivary Gland Neoplasms, salivary gland cancer, DNA-repair, biomarkers, prognosis.

## Introduction

Salivary gland tumorigenesis is a poorly understood process. In contrast with oral squamous cell carcinoma (OSCC), which has tobacco as a well establish risk factor and TP53-mutation as a recognized molecular-driven event (1), the extrinsic or/and intrinsic triggers of salivary gland tumors (SGT) are not clear. Progressively, distinctive chromosomal rearrangements or translocations are being uncovered in benign and malignant SGT (2). Yet, many key molecular events remain to be better understood in salivary gland tumorigenesis, such as deregulation in DNA-repair mechanisms.

Accurate DNA replication is crucial to maintain cell integrity at the genomic level. Malfunction of misincorporation repair mechanisms, such as the DNA mismatch-repair (MMR) system, can prompt a spontaneous mutator phenotype (3). As an example, the inheritance of MMR genes germline mutations significantly increases gastrointestinal and endometrial adenocarcinomas predisposition in Lynch Syndrome (3). In this scenario, the lack of functional proteins from this system is associated with an increased risk of some cancers. Interestingly, our group found peculiar associations in head and neck tumors. The presence of MMR protein overexpression was associated with a poor clinical outcome in OSCC (4) and ameloblastoma (5). The central hypothesis for our previous findings is that increased expression of MMR proteins could be a consequence of genomic instability, triggering a more aggressive cell phenotype (4).

The MMR system is composed by 3 related, yet distinct, protein subunits: MutSα (hMSH2-hMSH6), MutSβ (hMSH2-hMSH3), and MutLa (hMLH1-hPMS2). The hMutSα complex is required for MMR pathway initiation and preferentially detects base-base mismatches and 1 or 2 nucleotides insertion/deletion mispairs. The hMutSβ complex preferentially recognizes larger insertion/deletion mispairs (6). As far as we are concerned, only hMLH1 and hMSH2 expression have been investigated in SGT, and the results were inconsistent (7-9). Whereas Ohki *et al*. (2001) found no differences in hMSH2 levels among benign and malignant tumors (7), Castrilli *et al*. (2002) observed a lower expression of this protein in benign tumors (8). Remarkably, hMSH3 and hMSH6 expression in SGT remain completely undetermined. Therefore, the present study aimed to uncover if MutSα (hMSH2-hMSH6) and MutSβ (hMSH2-hMSH3) proteins expression differ between the most prevalent benign and malignant SGT, and if these proteins can predict malignant SGT behavior during follow-up.

## Material and Methods

- Study design and patients’ samples

This transverse observational study was approved by the Human Research Ethics Committee (CAAE protocol number: 22751019.4.0000.5418). Cases of SGT diagnosed between 2006 and 2016 were retrieved from the pathology service of Santa Rita Hospital - Irmandade da Santa Casa de Misericordia de Porto Alegre, Porto Alegre, Rio Grande do Sul, Brazil and from a surgical pathology service in Cascavel, Paraná, Brazil. Inclusion criteria consisted of at least 70% of information available in the medical records and availability of stored formalin-fixed paraffin-embedded samples. Exclusion criteria consisted of insufficient quantity of tumor area present in the specimens for tissue microarray construction.

Clinical data, including age, sex, and primary tumor site, were retrieved from medical files. For malignant SGT, clinical stage at diagnosis [based on the AJCC 8th classification (10)] and follow-up information were also retrieved. The follow-up period was defined as the time between diagnosis until the recurrence date (for disease-free survival) and death date (for disease-specific survival) or last visit to the hospital. Perineural invasion was assessed in all malignant SGT. All cases diagnosed as Adenoid Cystic Carcinoma (ACC) were classified according to the histopathological pattern in cribriform, tubular, or solid (11). Mucoepidermoid Carcinoma (MEC) cases were graded according to the criteria proposed by the Armed Forces Institute of Pathology – AFIP in low, intermediate, or high grade (12).

- Tissue Microarray Construction

Two trained oral pathologists (FPF and VPW) constructed the tissue microarray (TMA) blocks from the SGT specimens retrospectively collected. The methods used have been previously described and validated (13,14). Tumor areas from the central and most cellular zone of each SGT were selected. The pathologists were blinded to all clinical information during TMA construction and the tumor areas were selected using an objective marker all in the same day to avoid selection bias resulting from different time point analysis. A manual tissue arrayer (Sakura Co, Japan) was used to puncture the paraffin blocks at the corresponding area with a 2.0-mm needle. Three representative cylindrical cores were taken from each donor block and arranged sequentially in a 60-core ready-to-use recipient paraffin block (Sakura Co, Japan). A map specifying each case's precise location was made.

- Immunohistochemistry

Immunohistochemical reactions were carried out in 3-µm histologic sections of TMAs and NSG samples. Antigen retrieval was performed using citrate solution (pH 6.0) in an electric pressure cooker for 15 minutes. The slides were incubated overnight with primary antibodies against hMSH2 (Polyclonal; Santa Cruz Technology, CA; diluted 1:100), hMSH3 (Polyclonal rabbit antihuman; Novus Biologicals, Newcastle, UK; diluted 1:100), and hMSH6 (Polyclonal; Cell Marque, Rocklin, CA; diluted 1:50). Positive reactions were detected using the chromogen substrate diaminobenzidine tetrahydrochloride (DAB; Sigma, St. Louis, MO). Colon samples were used as positive control sections, and negative controls were obtained by omitting the primary antibodies.

- Digital Analysis

Immunostained slides were scanned into high-resolution images using the Aperio Scanscope (Aperio Technologies Inc, Vista, CA). Quantification was performed on .svs files using the "Nuclear V9" algorithm with the following parameters: average radius: 0.9; curvature threshold: 2.5; lower threshold: 0; upper threshold: 230; minimum nuclear size: 22; maximum nuclear size: 165; minimum roundness: 0.3; minimum compaction: 0.1; minimum elongation: 0.2; and an intensity threshold ranging from 0 to 230, in which the strong staining was considered from 0 to 185 and the weak staining was from 185 to 230. Each case was initially evaluated at lower magnification to identify the areas with a higher percentage of positive cells (hotspots). Next, the percentage of positive cells was determined, with at least 1000 cells being quantified in 10 hotspot areas. Our group has previously used the same formula in studies assessing MMR-proteins in OSCC (4) and odontogenic tumors (5).

- Statistical Analysis

Descriptive data were reported for clinic-demographic characteristics. hMSH2, hMSH3 and hMSH6 presented a non-parametric distribution. Therefore, the association of MMRS proteins and SGT diagnosis and histological features were carried by non-parametric tests such as Mann-Whitney and Kruskal-Wallis tests followed by Tukey post-hoc test. Spearman's correlation coefficients were calculated to determine the correlation between proteins of the complex MutSα (hMSH2-hMSH6) and MutSβ (hMSH2-hMSH3). Receiver Operating Characteristic (ROC) curves were constructed to establish the best cut-off point using tumor recurrence as the main outcome. The cut-off point was determined as the value presenting the highest sensitivity along with good specificity. Protein expression was then dichotomized into high or low expression. Next, cases with high expression of hMSH2 and hMSH6 simultaneously were classified as MutSαhigh, and cases with high expression of hMSH2-hMSH3 simultaneously were classified as MutSβhigh. Univariate survival analysis (for disease-free survival) was carried out with a Cox proportional hazards model. Kaplan-Meier cumulative survival curves were constructed and compared using the log-rank test. The analyses were performed in SPSS software (IBM Corporation, Armonk, NY), version 20.0, and GraphPad Prism (GraphPad Software, San Diego, CA). For all tests, p≤0.05 was considered indicative of statistical significance (NS – not significant; * *p*<0.05; ** *p*<0.01; *** *p*<0.001, **** *p*<0.0001).

## Results

- SGT sample characterization

In total, 102 cases were included in this study, comprising 10 cases of NSG, 37 cases of pleomorphic adenoma (PA), 17 cases of Warthin tumor (WT) (total of 54 benign SGT), 19 cases of MEC, and 19 cases of ACC (total of 38 malignant SGT). Table 1 shows the main demographic characteristics of patients according to each diagnosis.

**Table 1:**
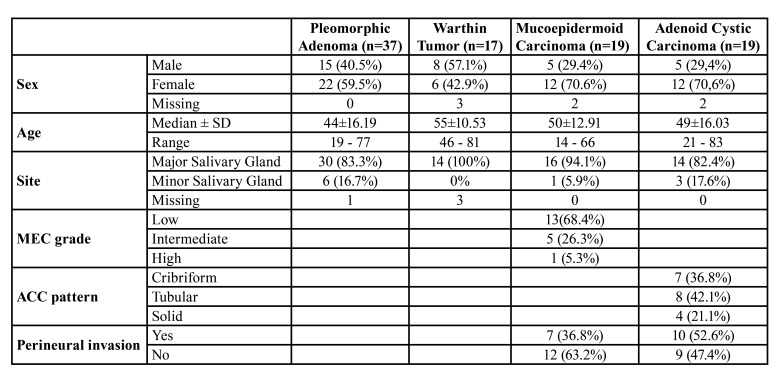
Clinic-demographic features of SGT.

MEC were classified as low-grade in 13 cases, intermediate grade in 5 cases, and high-grade in 1 case. For statistical analysis, intermediate and high grades were grouped due to the limited number of cases. In ACC, 7 cases were classified histologically as cribriform, 8 as tubular, and 4 as solid. Malignant tumors were also evaluated for the presence of perineural invasion (PNI). Seven MEC cases (36%) and 10 cases of ACC (52%) presented PNI. Mean follow-up of patients with malignant SGT was 44.9 months (range 0 to 130 months). During this period, six patients (2 cases diagnosed as MEC and 4 cases diagnosed as ACC) presented recurrence of disease. The average time for recurrence was 14 months, ranging from 1 to 87 months. Disease-free survival (DFS) rates for 2-years and 5-years were 88% and 79%, respectively. Only two patients died due to the tumor during follow-up. Both patients were diagnosed with ACC, and the deaths occurred 5 and 15 months after the diagnosis. Hence, the disease-specific survival (DSS) rates for 2-years and 5-years were the same, at 91%.

- hMSH2, hMSH3 and hMSH6 analysis according to diagnosis

**Figure 1:**
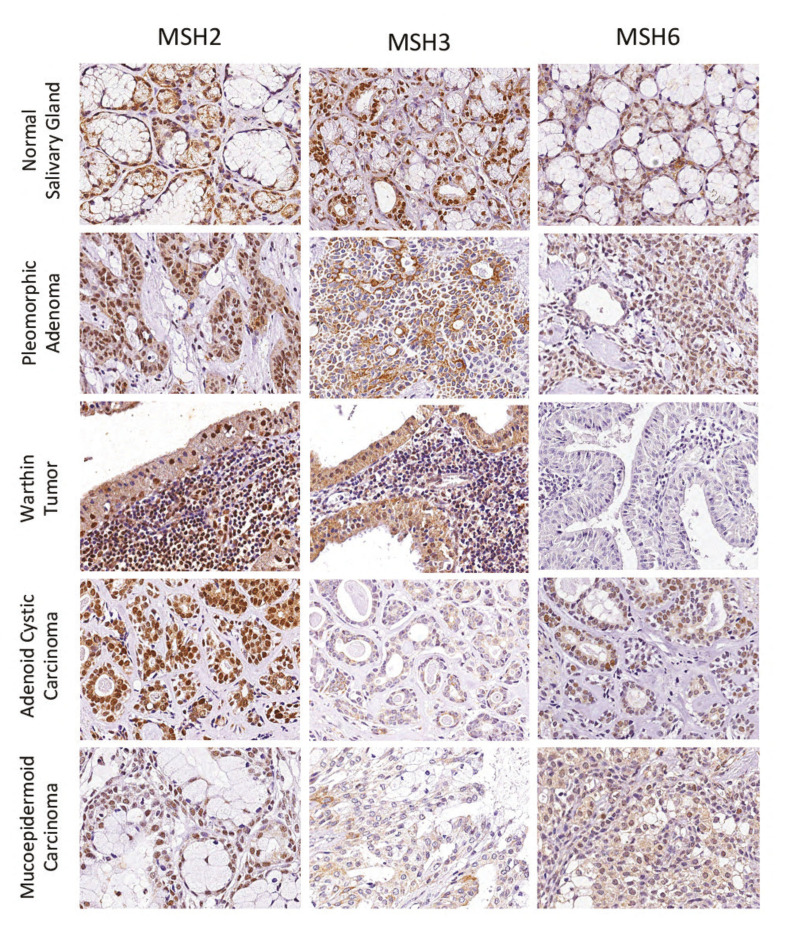
Representative images of hMSH2, hMSH3, and hMSH6 immunoexpression in Normal Salivary Glands, Pleomorphic Adenoma, Warthin Tumor, Adenoid Cystic Carcinoma, and Mucoepidermoid Carcinoma.

Representative images of hMSH2, hMSH3, and hMSH6 in NSG and SGT are presented in Fig. 1. According to SGT diagnosis, the mean, standard deviation, median, and range of positivity for all markers are shown in Table 2.

**Table 2:**
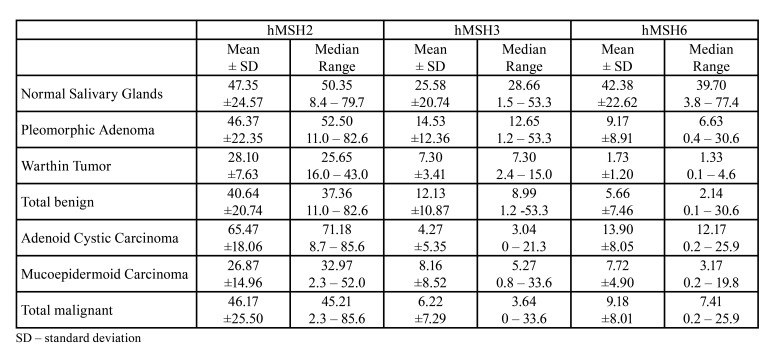
Percentage of positive cells to hMSH2, hMSH3, and hMSH4 according to SGT diagnosis.

All cases of NSG and SGT analyzed were positive for hMSH2. ACC presented the highest mean of hMSH2 positive cells [65.47], while WT presented the lowest (28.10) (Supplement 1). Statistical analysis revealed significant differences among PA vs ACC (*p*<0.05), PA vs MEC (*p*<0.05), WT vs ACC (*p*<0.0001) and ACC vs MEC (*p*<0.0001) (Supplement 1). The overall percentage of cells marked with hMSH2 was similar between NSG, benign, and malignant SGT (Supplement 1). No significant difference was observed concerning the expression of hMSH2 according to MEC grade and ACC histological pattern (Mann-Whitney test, *p*=0.96; Kruskal-Wallis test, *p*=0.64, respectively). Similarly, no differences were observed concerning the presence of PNI in MEC and ACC (Mann-Whitney test, *p*=0.65, and *p*=0.44, respectively).

All NSG and most of SGT were positive for hMSH3, however, with a significantly lower expression than hMSH2. Also, one case of ACC was negative for hMSH3. ACC stood out as the tumor with the lowest mean of hMSH3 expression [4.27], and PA presented the highest mean [14.35] among the SGT analyzed (Supplement 2). Differences were encountered for NSG vs ACC (*p*<0.01) and PA vs ACC (*p*<0.001) (Supplement 2). Moreover, malignant SGT showed reduced hMSH3 expression compared to NSG and benign SGT (Supplement 2). According to MEC grade and ACC pattern, there was no significant difference for hMSH3 expression (Mann-Whitney test, *p*=0.63, Kruskal-Wallis test, *p*=0.69 respectively). Despite the lack of a statistically significant result, we observed a tendency of lower hMSH3 expression in solid ACC cases (mean 2.5) compared to cases classified as cribriform (mean 4.9) and tubular (mean 4.5). The percentage of hMSH3 positive cells was similar among MEC cases with and without PNI (Mann-Whitney test, *p*=0.97). In ACC, however, cases with PNI presented a significant lower percentage of hMSH3 positive cells [2.05] compared to cases without invasion [6.75] (Mann-Whitney test, *p*=0.03).

Like hMSH3, hMSH6 was also expressed in a lower proportion of cells compared to hMSH2. For this protein, NSG stood out as presenting the higher percentage of positive cells, with a mean of 22.6%. WT and MEC presented the lowest values, with a mean of 1.73% and 7.72%, respectively. Significant differences were observed comparing NSG and all SGT (*p*<0.001), excepting ACC (Supplement 3). ACC also overexpressed hMSH6 compared to MEC and WT (Supplement 3). Overall, we detected that this protein was less expressed in benign and malignant SGT than NSG (Supplement 3). No significant difference was observed concerning the expression of hMSH6 according to MEC grade and ACC histological pattern (Mann-Whitney test, *p*=0.52; Kruskal-Wallis test, *p*=0.82, respectively). Similarly, no differences were observed concerning the presence of PNI in MEC and ACC (Mann-Whitney test, *p*=0.31, and *p*=0.65, respectively).

- MutSβ analysis

**Figure 2:**
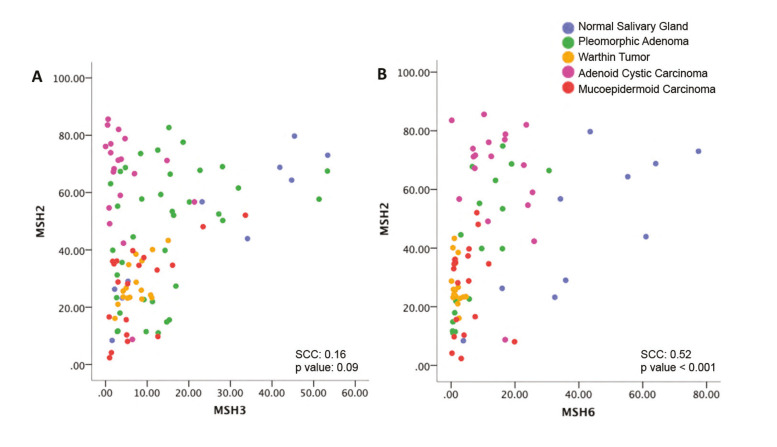
(A) Scatter plot of MutSβ complex (hMSH2 and hMSH3). (B) Scatter plot of MutSα complex (hMSH2 and hMSH6). Spearman's correlation coefficient and *p* values are detailed for each correlation considering all diagnoses combined.

There was no significant correlation between the expression of hMSH2 and hMSH3 when all cases were analyzed combined (SCC=0.16, *p*=0.09) (Fig. 2). However, when each diagnosis was evaluated individually (Supplement 4), significant direct correlations were identified for cases of NSG (SCC=0.95, *p*>0.0001), WT (SCC=0.53, *p*=0.02), and MEC (SCC=0.48, *p*=0.03), indicating that in these tumors an increase in hMSH2 is related to a concomitant increase on hMSH3 levels.

In PA cases, we observed a strong tendency towards a direct correlation, but the result was not statistically significant (SCC=0.32, *p*=0.05). Interestingly, ACC was the only tumor that showed a trend to an inverse correlation (SCC=-0.40, *p*=0.08).

- MutSα analysis

MutSα was assessed by evaluating hMSH2 and hMSH6 correlation. There was a significant direct correlation in the overall sample (Spearman's correlation coefficient (SCC=0.52, *p*<0.001) (Fig. 2). By analyzing each diagnosis individually (Supplement 4), it was possible to observe that this correlation occurred mainly due to a significant and robust correlation for cases of NSG (SCC=0.79, *p*=0.006) and PA (SCC=0.84, *p*<0.001). All other diagnoses showed no significant association, and by analyzing the scatter plots and SCCs, a trend of inverse correlation for WT and ACC was noticed (Supplement 4).

- Disease-free survival analysis of malignant SGT

**Figure 3:**
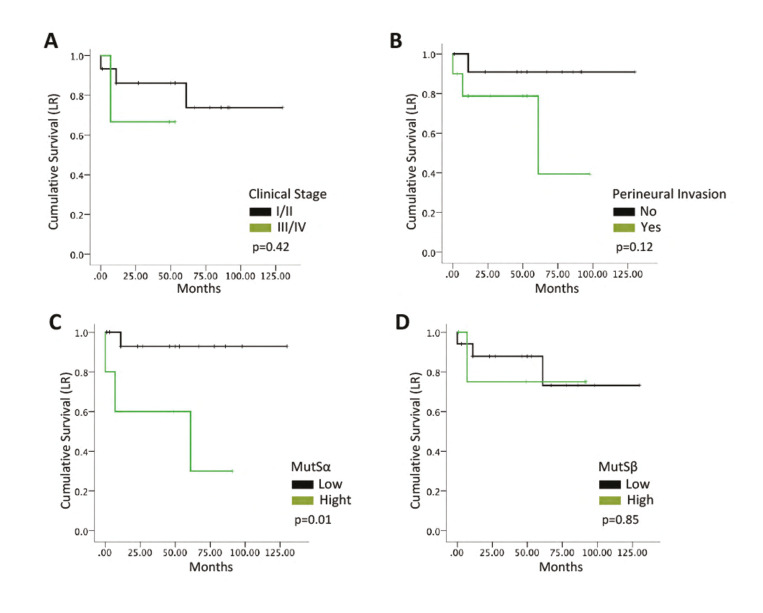
Kaplan-Meier cumulative survival curves for disease-free survival according to (A) Clinical Stage, (B) Perineural Invasion, (C) MutSα expression and (D) MutSβ expression (*p* values of the log-rank test).

Malignant SGT were further analyzed to determine the prognostic value of MMR proteins. ROC curves were constructed using recurrence as the main outcome and based on the analysis, cut-off values for hMSH2, hMSH3, and hMSH6 were determined as 48.6, 2.35, and 10.9, respectively. Among malignant cases, 47% were classified as hMSH2high, 63% as hMSH3high, and 34% as hMSH6high. Next, cases were dived in MutSαhigh (23.7%) and MutSβhigh (23.7%). Cox's univariate regression test (Table 3) revealed that only MutSα expression was associated with poor disease-free survival and that patients presenting MutSαhigh expression had a 10.26-fold increased risk of presenting local recurrence during follow-up compared to patients with MutSαlow. Using Cox's regression model, we also investigated PNI and TNM stage as prognostic factors. However, both factors were not associated with DFS in the present sample (PNI – *p*=0.16; TNM – *p*=0.43). Survival curves were constructed and compared using the log-rank test (Fig. 3), which corroborated with the Cox Regression results.

**Table 3:**
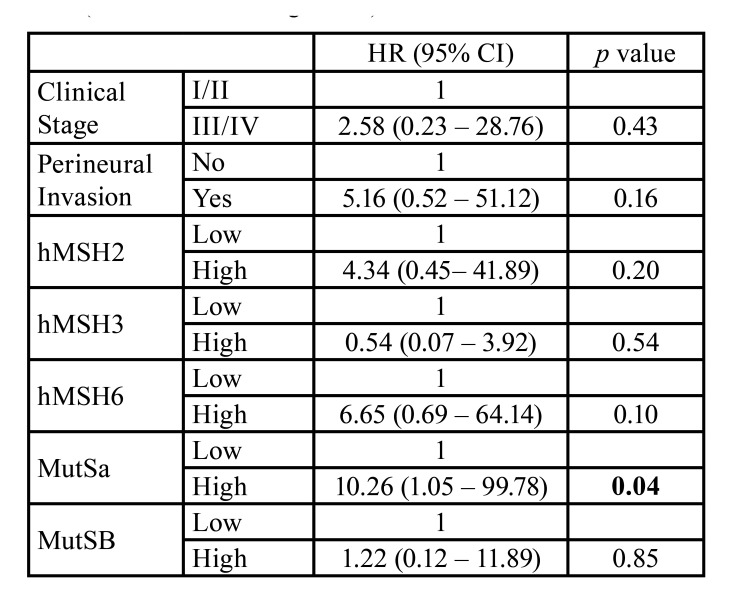
Independent variables association with disease-free survival (Univariable Cox Regression).

## Discussion

SGT are acknowledged as some of the most morphologically heterogeneous human neoplasms (15). In the present study, we aimed to unveil the role of MutSα and MutSβ associated proteins in SGT. To the best of our knowledge, this is the first time these two complexes of the MMR system were analyzed in a comprehensive sample of the most prevalent benign and malignant SGT, and results were also compared with NSG. Moreover, follow-up data available for the malignant cases allowed us to investigate the clinical relevance of deregulations in these proteins. Our most remarkable results demonstrated that hMSH3 is significantly downexpressed in malignant SGT compared to NSG and benign cases. The lack of hMSH3 also seems to be associated with a more aggressive phenotype, as it was associated with perineural invasion in ACC cases. Additionally, we could establish the clinical relevance of MutSα deregulation that corroborated with our previous findings in other head and neck tumors. Malignant SGT patients with increased expression of MutSα had a 10.26-fold increased risk of presenting local recurrence.

Functional inactivation of MMR genes is associated with a lack of protein expression (16). Our results demonstrated that in NSG, a reasonable percentage of cells were positive for all hMSH proteins analyzed, suggesting that MutSα and MutSβ are strongly expressed in physiological conditions. The rate of positive cells to hMSH2 was the highest (median 50.3%), followed by hMSH6 (median 39.7%), and finally hMSH3 (median 28.6%). Interestingly, hMSH2 expression, which was considerably elevated in NSG, was also equally increased in benign and malignant SGT. Among the proteins analyzed herein, hMSH2 was the only previously investigated by other groups. Ohki *et al*. (2001) found no differences in hMSH2 levels among benign and malignant tumors (7). Yet, these results were based on a cut-off for cellular positivity determined by the presence of more than 11% of neoplastic cells presenting reactivity to hMSH2. Thus, 100% of the sample, comprising 18 benign STG (PA and WT) and 16 malignant SGT (ACC, MEC, acinic cell carcinoma, and carcinoma ex-pleomorphic adenoma), was considered positive (7). hMSH2 was also investigated in SGT by Castrilli *et al*. (2002), which performed their analysis based on the percentage of positive cells, like our methods (8). The authors observed a mean of positive cells of 56.1 in malignant tumors compared to 31.1 in benign neoplasms (8).

Interestingly, the breakdown analysis by diagnosis also revealed that ACC presents the higher percentage of hMSH2 positive cells, which was 66.8 in their study, corroborating with our results. Herein, the mean percentage of hMSH2 positivity in ACC was 65.47. Nonetheless, we also observed disagreements, such as the values we obtained for hMSH6 positivity in PA and MEC (46.7 and 26.8, respectively) compared to theirs (31.1 and 47.8). We believe these differences might be associated with sample size and intrinsic heterogeneity of hMSH expression in SGT. For example, we analyzed 19 cases of MEC, while Castrilli *et al*.. (2002) only included 4 cases (8). Also, in most of our analyses, the standard deviation was considerably high, which implicates a relevant heterogeneity among tumors, even within the same subtype. Therefore, a representative sample is highly desirable for more accurate conclusions to be drawn. Our sample size was significantly larger than the previous samples used to investigate hMSH2 expression in SGT. Interestingly, when the results of hMSH2 of all SGT investigated herein is analyzed it is possible to detect that PA and ACC share a higher mean of positive cells while WT and MEC share a lower mean. It could be hypothesized that the different cell origin could influence this discrepancy, as PA and ACC share the same cell types and are mainly composed by luminal and myoepithelial cells while WT and MEC share the presence of epithelial cells originating for the more terminal part of the ductal system (striated and excretory duct). Further studies are necessary to investigate this assumption.

We found that hMSH3 protein was the only one that significantly differed between benign and malignant SGT. Malignant tumors presenting a mean of positive cells lower than benign tumors suggests that lack of hMSH3 expression might be associated with malignancy in salivary gland pathology. The malfunction of this protein can be associated with a more aggressive phenotype. We also observed that ACC cases with perineural invasion presented a lower percentage of hMSH3 positivity cells. Since this is the first analysis of hMSH3 in SGT, it is impossible to compare our results with similar studies. Yet, by looking at other malignancies or head and neck tumors, it is possible to find results that corroborate with our hypothesis. Kawakami *et al*. (2004) observed that hMSH3 immunohistochemical expression was inversely correlated with histological grade in bladder cancer: the higher and more aggressive the grade, the lower the percentage of hMSH3 positive cells (17). In accordance, both protein and mRNA levels of hMSH3 are decreased in advanced nasopharyngeal carcinoma (T3/T4) compared to early-stage tumors (T1/T2) (18). *In vivo* studies have demonstrated that in a background of hMSH6 deficiency, a further loss of hMSH3 accelerates tumorigenesis – supporting its importance as a tumor suppressor gene (19).

In our analysis, ACC stood out as the tumor with the highest percentage of hMSH2 cells, significantly different from MEC. Also, ACC had the lowest percentage of hMSH3 cells compared to all other tumors. Withal, while the correlation coefficients of MutSα analysis were positive - indicating a direct correlation - in all other SGT, in ACC, the value was negative – indicating an inverse correlation. This pattern of inverse correlation was also observed for ACC in the MutSβ analysis. These results suggest that while it seems to be overall deregulation in the MMR system in SGT, this appears to be further disturbed in ACC.

Recent studies using cutting-edge technology have uncovered that ACC has a relatively sTable genomic profile - Stephens *et al*. (2013) observed an average of 13 mutations per exome in ACC, which is considerably lower compared to the most common types of carcinomas (20). In this study, a few genes involved with DNA repair were found mutated, such as ATM, ERCC2, TP53, and CHEK2, yet in a very low percentage of cases (20). Similarly, Ho *et al*. (2013) observed some mutations in genes involved in DNA damage response in a panel comprising 55 ACC cases, such as TP53, UHRF1, TXNIP, ATM, BRCA1, and DCLRE1A (21). Interestingly, no mutations in the MMR-related genes were observed in both studies. Thus, we hypothesize that the MMR deregulation observed in ACC might result from epigenetic events, probably associated with methylation status. Previous studies have demonstrated that promoter methylation can trigger hMSH3 inactivation at the mRNA and protein levels in nasopharyngeal carcinoma (18). hMSH3 inactivation through promoter methylation is frequently observed in esophageal carcinoma – present in 91% of tumors compared to 76% in adjacent normal esophageal tissue (22). There are no studies evaluating the methylation status of MMR related genes in ACC and other SGT as far as we are concerned.

In the present study, we also observed that MutSα expression might have a prognostic impact on disease-free survival in malignant SGT. Our survival analysis included markers of tumor aggressiveness such as clinical stage and perineural invasion, and curiously both were not significantly associated with recurrence. At the same time, patients presenting MutSαhigh expression presented a 10.26-fold increased risk of presenting disease relapse. The lack of association of TNM stage and perineural invasion with prognosis could result from a rather small sample size of patients presenting recurrence. This limitation needs to be acknowledged. Remarkably, significant results of MutSα were achieved even in this scenario, and we observed an excellent curve separation in the Kaplan-Meier analysis. This result needs to be confirmed in larger samples, yet it corroborates precisely with our previous findings in head and neck squamous cell carcinoma and ameloblastoma (4,5). We hypothesize that MutSα increased expression is associated with a poor prognosis due to genomic instability that triggers this first response in the MMR system (4). Poor outcome might be a result of deficiencies in repairing this damage due to malfunction in other MMR proteins (such as hMSH3, as found herein). Other mechanisms are thought to play a role, and different DNA damage repair systems or evasion to apoptosis pathways are probably involved.

## Conclusions

SGT presents deregulations in MMR-associated proteins compared to normal salivary gland tissue. Remarkably, a lack in hMSH3 seems to be associated with a more aggressive phenotype (malignancy and perineural invasion), while increased activation of MutSα is associated with disease-free survival. Overall, our findings indicate that increased activation of MutSα complex is associated with a poor clinical outcome in head and neck tumors originating from salivary glands.
